# Internet addiction and depressive symptoms: a dose-response effect mediated by levels of physical activity

**DOI:** 10.47626/2237-6089-2021-0279

**Published:** 2023-02-02

**Authors:** Lauro Miranda Demenech, Marlos Rodrigues Domingues, Rosângela Mattos Muller, Vera Regina Levien, Samuel Carvalho Dumith

**Affiliations:** 1 Programa de Pós-Graduação em Ciências da Saúde Universidade Federal do Rio Grande Rio Grande RS Brazil Programa de Pós-Graduação em Ciências da Saúde, Universidade Federal do Rio Grande, Rio Grande, RS, Brazil.; 2 Programa de Pós-Graduação em Educação Física Universidade Federal de Pelotas Pelotas RS Brazil Programa de Pós-Graduação em Educação Física, Universidade Federal de Pelotas, Pelotas, RS, Brazil.; 3 Departamento de Saúde Materno-Infantil Universidade Federal de Pelotas Pelotas RS Brazil Departamento de Saúde Materno-Infantil, Universidade Federal de Pelotas, Pelotas, RS, Brazil.

**Keywords:** Internet addiction, depressive disorder, addictive behavior, physical activity, students

## Abstract

**Introduction:**

There are several negative impacts on the physical and mental health of people presenting internet addiction, including development of mood disorders, such as depression.

**Objective:**

The aim of the study was to evaluate the association between internet addiction and depressive symptoms, as well as to test the mediating role of physical activity in this association.

**Methods:**

A cross-sectional study was conducted with undergraduate students from three universities (one private and two public institutions) in southern Brazil. Depressive symptoms were measured with the Beck Depression Inventory (BDI-21), internet addiction with the Internet Addiction Test (IAT-20), and physical activity with the International Physical Activity Questionnaire (IPAQ – short version). Poisson regression and the Karlson-Holm-Breen mediation method were used for statistical analyses.

**Results:**

We observed a dose-response relationship between internet addiction and depressive symptoms. Levels of physical activity mediated the association between moderate internet addiction and depressive symptoms, accounting for 10.7% of the effect observed.

**Conclusion:**

Internet addiction can be detrimental to individuals’ health, contributing to development of depressive symptoms. Along with psychological and pharmacological therapies, prescription of physical activities is recommended.

## Introduction

Since its creation in the late 1960s, the internet has been gaining more and more space in our lives, allowing us to connect more easily and for longer than at any other time in human history. Recently, due to wireless technologies, and with the popularization of smartphones, access to the internet has become easier, changing the way we communicate and spread information, making the internet a ubiquitous part of everyday life and contemporary culture.^1^ It is a tool used by individuals of all ages; but younger people are more likely to use the internet with all its purposes and functionalities.^2^

It is indisputable that the internet has catalyzed development of society and its impacts echo in different contexts, such as education, health, politics, and communication, especially due to the exponential increase in the capacity to store and transmit information.^3^ However, this “symbiotic” relationship with the internet can also have negative effects. One example is internet addiction, which, despite not being recognized as a mental disorder in diagnostic manuals, has similar consequences to gambling and alcohol and drug abuse disorders.^4^ One example of this similarity is the neuroanatomical and neurochemical changes in the brain’s reward circuitry resulting from excessive internet use.^5^

The international literature has documented important negative impacts on the physical and mental health of people with internet addiction, including development of mood disorders, such as depression.^5 - 7^ Undergraduates seem to be a group especially susceptible to development of internet addiction, since studies indicate high prevalence of this outcome, ranging from 16.7%^8^ to 48.5%.^9^

Depression also seems to be highly frequent in this subgroup, considering that results from meta-analyses indicate that 28.5% of Brazilian^10^ and 30.6% of US university students^11^ have significant depressive symptomatology, proportions almost three-fold higher than among the general populations of both countries.^11 , 12^ Moreover, evidence from distinct cultural contexts indicates that the association between internet addiction and depression is very consistent among undergraduate students.^6 , 7 , 9 , 13^

However, the mechanisms that explain this association are less clear.

One plausible hypothesis is abandonment of habits known to be healthy for human development. For instance, physical activity has long been recognized as a protective factor against several mood disorders, such as depression,^14 , 15^ and there is evidence that levels of physical activity tend to be lower among individuals with internet addiction.^6 , 7^ Undergraduate students report low levels of physical activity,^16^ mainly because of lack of time due to a busy academic schedule.^17^ Insufficiency or absence of physical activity among subjects with internet addiction may have psychological and physiological impacts that increase the likelihood of developing depressive symptoms.

Although the high burden of depressive symptoms and the low levels of physical activity among undergraduates are well documented, internet addiction is a more recent phenomenon.^18^ The complex interrelationships between these three variables are becoming an emergent issue. Simultaneous occurrence of internet addiction and depression may be extremely detrimental to the patient and a major challenge for mental health experts. Identification of possible mechanisms underlying this association can help professionals managing these situations in their decision-making processes. Therefore, this study aimed to measure the prevalence of internet addiction and depressive symptoms in a sample of Brazilian university students and analyze the association between these two outcomes and also aimed to test the hypothesis that physical activity levels play a mediating role in this association.

## Methods

### Study design, locations, and participants

A cross-sectional study was carried out in southern Brazil with freshmen undergraduates from three universities (Universidade Federal de Pelotas, Universidade Católica de Pelotas, and Instituto Federal Sul-rio-grandense). Data collection took place from August 2016 to March 2017 and was carried out using anonymous, self-administered questionnaires.

### Sample

A calculation was performed to estimate the minimum number of participants for this study. This sample size calculation indicated that it would be necessary to sample 1,023 individuals in order to investigate associations with the following parameters: ratio of exposed (presence of internet addiction) to unexposed (absence of internet addiction) of 1:4, prevalence ratio of depressive symptoms of 2.0, 80% power, and 5% significance level, plus an additional 10% for possible losses and refusals and another 15% to control for confounding factors.^19 , 20^ Students were randomly sampled from each university, considering the proportionality of the expected number of freshmen enrolled at 2016.

### Variables and instruments

The outcome was presence of depressive symptoms and was evaluated with the Beck Depression Inventory (BDI), a self-perception instrument including 21 items, each scored from zero to three. Total scores above 12 are considered positive for depressive symptoms. The Inventory has been validated in Portuguese with clinical samples and its utility as a measurement of depressive symptoms has been established in a non-clinical population (Cronbach’s alpha = 0.81).^21^

The main exposure was internet addiction and was evaluated with the Internet Addiction Test (IAT), a self-administered 20-item questionnaire with Likert-type response options, validated in Brazil (Cronbach’s alpha = 0.90).^22^ Each item is scored from 1 (rarely) to 5 (always), with a maximum total score of 100 points. Respondents presenting scores below 30 were considered free from internet addiction, those with scores from 31 to 49 were mildly addicted, those with scores from 50 to 79 were moderately addicted, and those scoring over 80 were severely addicted.

To enable inclusion of levels of physical activity as a potential mediator, we measured physical activity with the short version of the International Physical Activity Questionnaire (IPAQ), validated in Brazil (correlation coefficient = 0.74).^23^ It contains eight questions about time spent in different activities per week, such as walking and moderate and vigorous physical activity. Individuals were categorized as inactive (0 minutes/week), insufficiently active (1 to 149 minutes/week), or active (≥ 150 minutes/week) for descriptive purposes. For the mediation analysis, this variable was operationalized in its numerical format (i.e., total time spent performing physical activities/week).

Data on socioeconomic and demographic characteristics were also collected as independent variables, namely: sex (male/female), age (18-19/20-25/26 years or more), skin color (white/black, brown, and yellow), university (Universidade Católica de Pelotas/Universidade Federal de Pelotas/Instituto Federal Sul-rio-grandense), Brazilian socioeconomic classification (A/B1/B2/C, D, and E), alcohol consumption (no/yes), and smoking (no/yes). Lastly, , self-reported information about weight and height were obtained and used to calculate body mass index, which was then categorized as underweight (< 18.5 kg/m^2^), healthy weight (18.5 - 24.9 kg/m^2^), or overweight and obesity (≥ 25.0 kg/m^2^).

### Statistical analysis

We used descriptive statistics to measure internet addiction and depressive symptoms. Bivariate analyses were performed to measure the distribution of outcomes according to independent variables. A multivariate analysis (Poisson regression with robust adjustment of variance^24^ ) was carried out to control for potential confounders (sex, age, socioeconomic level, skin color, university, alcohol consumption, smoking, physical activity, and body mass index). Later, a mediation analysis was conducted to assess whether the association between internet addiction (mild and moderate) and depressive symptoms could be mediated by physical activity levels, also controlling for confounding effects (i.e., variables with p ≤0.2 in the multivariate analysis). We chose the Karlson-Holm-Breen method,^25^ which breaks down the total effect of the exposure (internet addiction) on the outcome (depressive symptoms) into direct and indirect effects, considering potential mediation variables (physical activity levels). Using this technique, we were able to obtain the magnitude of the indirect effect and the proportion of total association explained by the pathway hypothesized. Analyses were conducted with the statistical package STATA 14, and the confidence level was set at 5%.

### Ethics

All students signed an informed consent form. Those with positive screening tests for internet addiction and depressive symptoms were referred to the outpatient clinics of their respective institutions. The study was approved by the Ethics Committee at the Universidade Católica de Pelotas under appraisal number 56053616.2.0000.5339.

## Results

We analyzed data from 1,026 undergraduates, mostly females (59.9%), with white skin color (81.0%), aged between 18 and 25 years (81.9%). Two thirds of them were from public universities, nearly 40% reported a family income of up to US$ 450 per month, and the majority were classified as socioeconomic classes B1 to E (62.0%). Three out of four students reported alcohol consumption, 9.9% were smokers, 9.4% were obese, and 12.1% were physically inactive ( Table 1 ). Cronbach’s alpha coefficients for the BDI, IAT, and IPAQ results for the sample under study were 0.90, 0.87, and 0.72, respectively.

**Table 1:** Sample description and prevalence of mild or moderate Internet Addiction and depression by categories. Sample of 1,026 undergraduate students, Pelotas, Brazil, 2017

Category	n	%	Mild or moderate Internet addiction (%)	Depression (%)
Sex (n = 1,025)			p = 0.338	p = 0.027
Male	411	40.1	43.7	15.4
Female	614	59.9	40.6	21.2
Age (n = 1,017)			p < 0.001	p = 0.731
18 to 19 years	418	41.1	46.7	18.9
20 to 25 years	415	40.8	45.0	19.5
26 years or older	184	18.1	23.9	16.6
Skin color (n = 1,020)			p = 0.148	p = 0.003
White	826	81.0	40.6	17.0
Black, brown, or yellow	194	19.0	46.3	26.7
University (n = 1,025)			p = 0.837	p < 0.001
Universidade Católica de Pelotas (private)	353	34.4	40.9	12.1
Universidade Federal de Pelotas (public)	630	61.5	42.5	21.3
Instituto Federal Sul-rio-grandense (public)	42	4.1	39.0	35.1
Brazilian socioeconomic classification (n = 921)			p = 0.012	p = 0.136
A	350	38.0	46.8	15.9
B1	167	18.2	31.3	17.7
B2	238	25.8	42.6	23.7
C, D, or E	166	18.0	42.2	21.0
Alcohol consumption (n = 1,009)			p < 0.001	p = 0.343
No	243	24.1	31.2	21.0
Yes	766	75.9	45.2	18.1
Smoking (n = 1,026)			p = 0.527	p = 0.184
No	924	90,1	41.4	18.2
Yes	102	9,9	44.8	23.9
Body mass index (n = 981)			p = 0.118	p = 0.600
Underweight (< 18.5 kg/m^2^)	27	2.8	55.6	25.9
Healthy weight (18.5 to 24.9 kg/m^2^)	638	65.0	43.1	17.8
Overweight (25.0 to 29.9 kg/m^2^)	224	22.8	35.9	20.9
Obesity (> 29.9 kg/m^2^)	92	9.4	38.6	19.2
Physical activity in leisure time (n = 977)			p = 0.007	p < 0.001
Physically inactive (0 min/week)	118	12.1	52.2	33.3
Insufficiently active (1 to 149 min/week)	502	51.4	43.2	20.7
Physically active (≥ 150 min/week)	357	36.5	36.2	12.2
Total			**41.7**	**18.8**

n = valid responses by category; n = absolute frequency; % = relative frequency (prevalence); Private = private institution; Public = public institution.

Differences in proportions of internet addiction and depression according to study categories tested with Pearson’s Chi-square test.

Regarding internet addiction, prevalence rates of mild and moderate addiction were 34.2% and 7.5%, respectively (no cases of severe internet addiction were identified). There were no gender differences with respect to this outcome. However, it was more frequent among individuals who were younger (p < 0.001), were from socioeconomic class B1 (p = 0.012), reported alcohol consumption (p < 0.001), or had less physical activity (p = 0.007). The overall prevalence of depressive symptoms was 18.7% and was higher among females (p = 0.027), with no association with age. Nevertheless, depressive symptoms were more prevalent among individuals with black, brown, or yellow skin color (p = 0.003), from public universities (p < 0.001), and with less physical activity (p < 0.001) ( Table 1 ).

**Figure 1:**
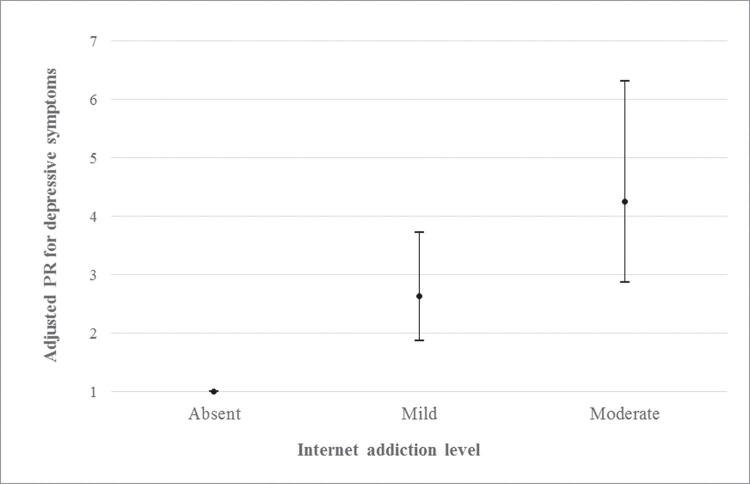
Prevalence ratios for depressive symptoms among individuals with mild and moderate internet addiction. Adjusted analysis controlled for sex, age, socioeconomic level, skin color, university, alcohol consumption, smoking, physical activity, and body mass index (see Table 2). Sample of 1,026 undergraduate students, Pelotas, Brazil, 2017.

Those with internet addiction were more likely to have depressive symptoms. After controlling for potential confounders, a dose-response effect was observed ( Table 2 ). More specifically, the adjusted prevalence ratio for depression among individuals with mild and moderate internet addiction was 2.64 (95%CI 1.87-3.73), compared to 4.26 (95%CI 2.87-6.31) for those without internet addiction (p < 0.001) ( Figure 1 ).

**Table 2:** Results of multivariate analysis of factors associated with depression conducted by Poisson regression with robust adjustment of variance. Sample of 1,026 undergraduate students, Pelotas, Brazil, 2017

Variable	PR (95%CI)	p-value
Internet addiction (mild)	2.64 (1.87-3.73)	p < 0.001
Internet addiction (moderate)	4.26 (2.87-6.31)	p < 0.001
Sex (female)	1.13 (0.84-1.52)	p = 0.402
Age (20 to 25 years)	1.07 (0.80-1.42)	p = 0.655
Age (26 years or more)	0.91 (0.57-1.47)	p = 0.708
Skin color (black, brown, or yellow)	1.19 (0.87-1.65)	p = 0.279
Brazilian economic classification (decrease in classification)	0.97 (0.91-1.05)	p = 0.509
University (public institution)	1.51 (1.02-2.23)	p = 0.042
Smoking (yes)	1.58 (1.01-2.48)	p = 0.046
Alcohol consumption (yes)	1.55 (1.25-1.93)	p < 0.001
Insufficiently physically active	1.40 (0.97-2.00)	p = 0.068
Physically inactive	2.11 (1.40-3.20)	p < 0.001
Body mass index (obesity)	0.82 (0.49-1.39)	p = 0.467

95%CI = 95% confidence interval; PR = prevalence ratio.

**Figure 2:**
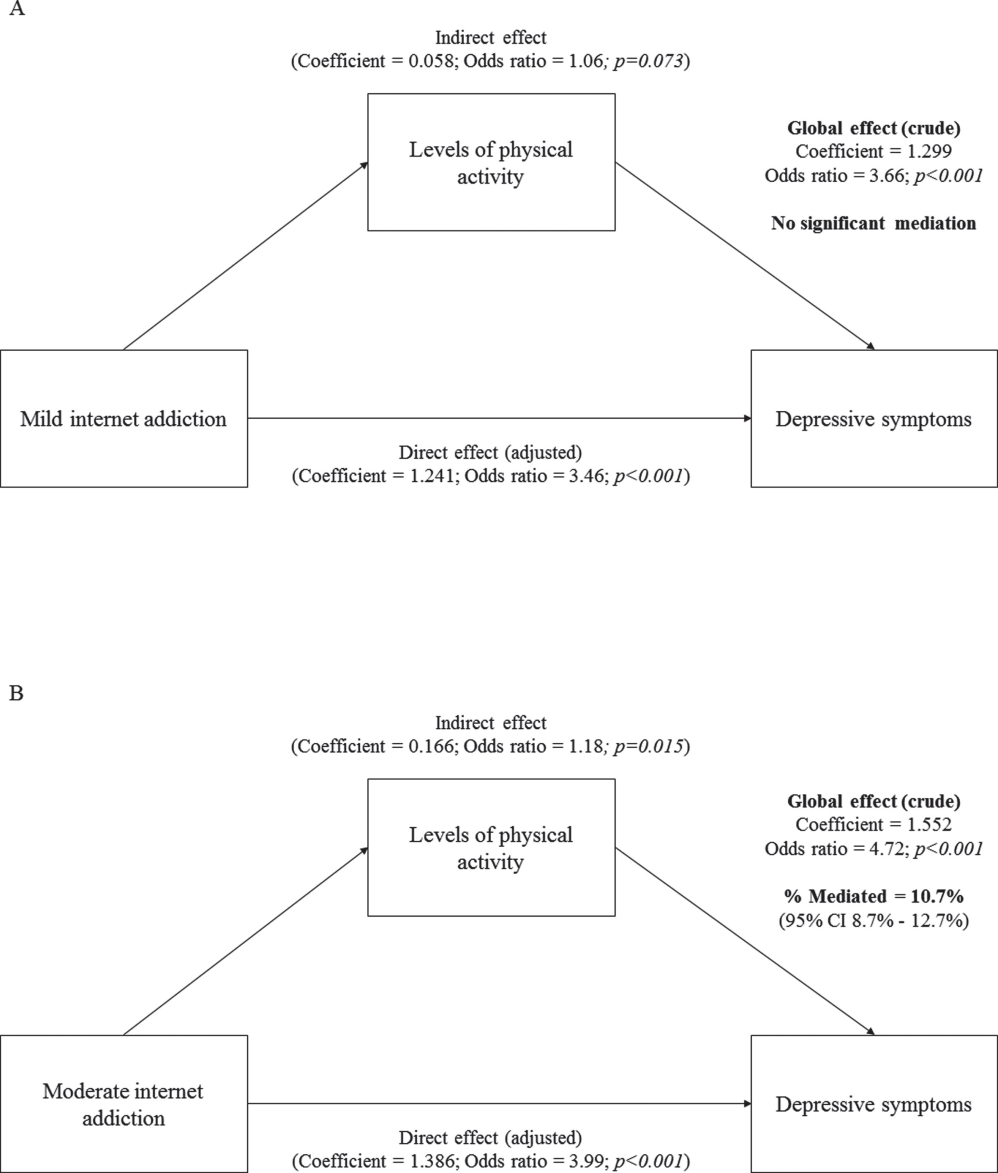
A) Global, direct, and indirect effects (coefficients, odds ratios, and p-values) between mild internet addiction and depressive symptoms, mediated by levels of physical activity. B) Global, direct, and indirect effects (coefficients, odds ratios, and p-values) between moderate internet addiction and depressive symptoms, mediated by levels of physical activity. Analysis adjusted by variables with p < 0.2 for their associations with depression (i.e., university type, alcohol consumption, and smoking; see Table 2). Sample of 1,026 undergraduate students, Pelotas, Brazil, 2017.

Lastly, after division into direct and indirect effects on the association between internet addiction and depressive symptoms, and controlling for possible confounders, we identified that levels of physical activity played a mediating role between moderate internet addiction and depressive symptoms – accounting for 10.7% (95%CI 8.7-12.7, p = 0.015) of the effect. This mediation was not observed between mild internet addiction and depressive symptoms ( Figure 2 ).

## Discussion

There are several ways to classify pathological use of the internet, which makes it difficult to compare different investigations.^4 , 26^ However, the prevalence of mild or moderate internet addiction in our study was 41.7%, which is higher than found in similar studies conducted in Pakistan (16.7%),^8^ Qatar (17.6%),^7^ Turkey (24.3%),^6^ and Japan (38.2%),^13^ but lower than reported by a Japanese study with undergraduates (48.5%).^9^ In addition, the prevalence of more severe cases of internet addiction in this sample (7.5%) was higher than in the Brazilian general population (4.8%).^27^ The sample under study is composed mainly of young individuals; a generation that has grown up in a world already fully connected by the internet, therefore constituting a subgroup with greater likelihood of internet addiction.^28^ The prevalence of depressive symptoms observed in this investigation (18.7%) seems to agree with what would be expected for this population. A multicenter study with 12,677 Brazilian undergraduates from public and private institutions, using the same instrument, reported a similar prevalence, with 18.6% of participants having depressive symptoms.^29^ However, this proportion was higher than among the Brazilian general population (14%).^12^ Undergraduate students have being recognized as a subgroup with a high frequency of depressive symptoms, due to the stressful academic background, excessive workload, and developmental stage susceptibility.^10^

A dose-response effect was observed between internet addiction and depressive symptoms. Other studies investigating internet addiction have also found an association with depressive symptoms.^6 , 7 , 13^ A study with Japanese undergraduates also reported a dose-response relationship between these two variables, controlling for confounding variables. People with moderate and severe internet addiction were, respectively, 2.9 and 7.3 times more likely to have depressive symptoms compared to those without internet addiction.^9^ In a longitudinal study, participants with higher internet addiction scores were more likely to develop mental health problems, such as depression.^30^ One hypothesis to explain this result is a vicious cycle between internet addiction and depression. People may use the internet to reduce stress, increasing internet use and, consequently, decreasing social contact. In turn, deterioration of interpersonal relationships may generate more stress, exacerbating depressive conditions, and making them seek even more relief from the internet.^9^

One important result of our research was the mediating role of levels of physical activity on the association between moderate internet addiction (but not mild addiction) and depressive symptoms. In other words, participants with a higher degree of internet addiction are more likely to report depressive symptoms, which was partly explained (10.7%) by lower levels of physical activity. No studies have been identified investigating physical activity as a potential mediator of this association. On the other hand, several studies have identified an association between internet addiction and low levels of physical activity,^6 - 8^ as well as between physical activity and depression.^14 , 31 , 32^

There is evidence to support this mechanism and its occurrence is both psychologically and biologically plausible. As previously mentioned, individuals can use the internet as a strategy to deal with various stressors. This habit may be perpetuated through negative reinforcement, since apparently it can be helpful to remove unpleasant stimuli, increasing the likelihood of being used in the future.^33^ This mechanism can lead to the vicious cycle mentioned above, causing people to use the internet excessively, further reducing their contact with real-world and face-to-face activities, such as physical activity. The higher the degree of internet addiction, the greater the lack of such behaviors – which can explain why levels of physical activity mediated the association between moderate internet addiction (but not mild) and depressive symptoms.

Furthermore, while physically inactive undergraduates are more likely to present internet addiction, they are less likely to report satisfaction with their sleep and more likely to feel stressed,^34^ two variables that are highly correlated with occurrence of depressive symptoms.^35 , 36^ Both sleep deprivation and chronic stress can disrupt neuroplasticity, precipitating or exacerbating depression.^35 , 36^ Considering that individuals with moderate internet addiction presented significantly lower levels of physical activity, they may therefore have worse sleep quality and higher levels of stress than their peers without internet addiction, which can contribute to explanation of the mediating role of physical activity in the issue discussed in this study.

There is a debate about the bidirectionality of the association between physical inactivity and depression. However, a large-scale study, using bidirectional analyses, of Mendelian randomization, only identified evidence for one direction of this association, in which physical inactivity has a possible causal relationship with the occurrence of depression (but not the other way around).^14^ Depressed people may present structural adaptations in the amygdala and prefrontal cortex and a reduction in the hippocampus volume, which could be reduced or prevented through neurogenesis resulting from regular physical activities.^31^

Levels of physical activity mediated 10.7% of the association between moderate internet addiction and depressive symptoms, suggesting that other direct and indirect effects may help to explain the causal pathway. Notwithstanding, this result is important, because it identifies a therapeutic path that can be stimulated by mental health professionals. It is estimated that there is a significant reduction in the risk of developing major depressive disorder with small changes, such as substituting sedentary behavior for 15 minutes of vigorous physical activity (such as running) or 1 hour of moderate physical activity (such as brisk walking).^14^ Physical activities can be effective in treating depression, especially if used in combination with psychotherapeutic and pharmacological interventions,^15 , 31^ and have good adherence rates (from 50% to 100%).^32^

Besides, engagement in physical activity can break the vicious cycle between internet addiction and depression. This can occur by exchanging the negative reinforcement “stress relief through the internet” for another mechanism, “stress relief through physical activity,” and by adding some positive reinforcement from physical activity (such as release of endorphins and serotonins and the feeling of performing an activity that is socially considered as healthy). Thus, the probability of eliminating the behavior of using the internet excessively is increased and depressive symptoms are reduced.^33^

Lastly, this study must be interpreted within its limitations. First, its cross-sectional design does not allow the temporality of events to be observed. Second, physical activity assessment was based on self-report measures, which can be influenced by mood states, such as depression. Third, the course taken and the shift of study of the undergraduates may constitute possible sources of confounding of the results, but these data were not collected. However, this is an innovative study, since no prior research was found that in addition to investigating the relationship between internet addiction and depression has also identified a mediating role of physical activity in this association.

## Conclusions

Therefore, we conclude that the prevalence of internet addiction and depressive symptoms in this sample was high, and that there is a dose-response relationship between them. The higher the levels of internet addiction, the greater the probability of depressive symptoms, even when controlling for potential confounders such as socioeconomic, demographic, and behavioral factors. Levels of physical activity mediated 10.7% of the association between moderate internet addiction and depressive symptoms, indicating that it may be an important therapeutic tool. A multiprofessional approach is recommended for treatment and monitoring of cases, considering the expertise required to prescribe and follow-up physical activities, psychotherapy, and/or pharmacotherapy.
